# Selenium Status, Its Interaction with Selected Essential and Toxic Elements, and a Possible Sex-Dependent Response In Utero, in a South African Birth Cohort

**DOI:** 10.3390/ijerph18168344

**Published:** 2021-08-06

**Authors:** Halina B. Röllin, Kalavati Channa, Bukola Olutola, Jon Øyvind Odland

**Affiliations:** 1 School of Health Systems and Public Health, Faculty of Health Sciences, University of Pretoria, Pretoria 0002, South Africa; Bukola.Olutola@gmail.com (B.O.); Jon.o.odland@ntnu.no (J.Ø.O.); 2 Environment and Health Research Unit, South African Medical Research Council, Johannesburg 2094, South Africa; 3 Department of Analytical Chemistry, Lancet Laboratories, Johannesburg 2090, South Africa; kalavati.channa@lancet.co.za; 4 School of Engineering, IT, Science and Health, Independent Institute of Education-Monash, Roodepoort 1724, South Africa; 5 Department of Community Medicine and Nursing, Faculty of Health Sciences, University of Science and Technology, N-7491 Trondheim, Norway; 6 Department of General Hygiene, I.M. Sechenov First Moscow State Medical University (Sechenov University), 119435 Moscow, Russia

**Keywords:** selenium in utero, essential elements, toxic elements, birth outcomes, sex-dependent response

## Abstract

Selenium (Se) is an essential trace element and its deficiency in utero may affect fetus development and birth outcomes. The current study aimed to assess serum Se status at delivery and examine the possible association between Se levels and birth outcomes. The interaction of Se with selected essential and toxic elements as well as possible sex-dependent responses in utero were also evaluated. The negative association between Se levels and head circumference of neonates was evident in the total cohort (β = −0.164; *p* < 0.001) as well as in the pre-term and full-term cohorts. Significant positive correlations were found between maternal serum Se concentrations and zinc (Zn) and copper (Cu) in the total and regional cohorts. In the total cohort, the toxic elements lead (Pb) and arsenic (As) showed a negative correlation with Se levels, while mercury (Hg), aluminum (Al) and cadmium (Cd) showed a positive correlation. The study found a sex-dependent response in utero for Zn, Cu, Pb, Hg, and Al. The findings of the current study may inform reproductive health policy on Se status in South Africa and highlight the need for sensitive methods to measure Se intake during pregnancy and its complex interactions with other micronutrients and environmental pollutants.

## 1. Introduction

Selenium (Se) is an essential trace element and its status in humans and other mammals depends primarily on dietary intake, with the main sources being meat, cereals, and seafood [1]. Globally, the intake of Se in the diet is affected by the geosphere that differs by geographical positions of the population [2]. Dietary Se deficiency is particularly evident in many regions where agricultural soils producing staple crops are naturally Se-deficient, necessitating bio-fortification of soils in some countries [3]. The U.S. Institute of Medicine (IOM) has the recommended daily allowance (RDA) for Se set at 55 µg for adults, with the RDA for pregnant women set at 60 µg [4]. The European Food Safety Authority (EFSA) set a reference intake of 70 µg/day for adult men and women based on the relationship between intake of Se and plasma selenoprotein P (SEPP1) concentration [5].

The levels of Se are highly regulated in biological systems and incorporated into selenoproteins. Se plays an integral role in immune function and antioxidant defense, with 25 genes expressing selenoproteins in the human genome [6,7,8,9]. Se is also involved in the metabolism of thyroid hormones and reproduction [10,11]. When present in adequate concentrations in human systems, and due to its enzymatic functions, Se has been shown to exert many beneficial health effects such as prevention of cardio- and neuro-defects, prevention of cancer, and increasing immune-competence and reproductive capacity [12,13,14,15,16].

Number of studies have shown that Se can easily be transferred from the mother to the fetus ([17,18,19]). The free trans-placental passage of Se indicates the strong dependence of the developing fetus on the Se levels of their mothers [20]. It has been reported that Se passes the placental barrier by a passive, diffusion-mediated mechanism across the placental membrane [21].

In humans, Se deficiency may affect the heart, weaken the immune system, and cause hypothyroidism [22,23,24]. For example, Keshan disease (cardiomyopathy disease of the heart muscle) and Kashan–Beck disease (a disorder of the cartilage and bone) are linked to Se deficiency [25]. On other hand, it has been shown that adequate levels of Se may reduce cardiomyopathy in women and children with Keshan disease [26,27]. Furthermore, Se deficiency is associated with sepsis, arteriosclerosis, cancer, cognitive decline, and higher mortality rate in older persons [28,29].

It is of concern that low Se status during pregnancy may result in adverse pregnancy outcomes such as miscarriages and premature birth, neural tube defects, low birth weight, and gestational diabetes [30,31,32]. Furthermore, many of these adverse pregnancy outcomes have been reported in women infected with the human immunodeficiency virus (HIV) and their offspring [33]. It is reported that in mothers with hypothyroidism, complications such as miscarriage and pre-eclampsia are common, as are impaired motor function in neonates and speech disorders in infants [34]. An excess of Se in humans, resulting mainly from environmental and accidental exposures or uncontrolled intake of Se supplements, may cause selenosis, a condition that presents with hypotension, tachycardia, gastrointestinal symptoms, neurological dysfunction and fetor oris (halitosis) [35].

It is understood that since Se is an essential trace metalloid, it plays a detoxification role in metal toxicity by mitigation of cadmium (Cd) and arsenic (As) induced tissue injuries [36,37,38]. It has been shown in animal models that Se also reduces the toxicity of mercury (Hg) by making it biologically inert through its binding to selenite or other Se-containing ligands. In humans, the Hg–Se interaction suggests a reduction in toxicity of both inorganic mercury (iHg) and methyl mercury (MeHg) forms, although it should be noted that these study findings appear to be inconsistent [39].

Due to the essential functions that Se plays in human health, many studies have been undertaken to evaluate Se status in the general population, in women of reproductive age, and in pregnant women. To date, there is a paucity of such investigations in the Southern Hemisphere and as far as the literature reviews inform, no investigations have been conducted into Se levels in pregnant women nor the general population in South Africa.

The current study assessed Se status in utero at delivery and examined the possible association between Se levels and birth outcomes in coastal populations in South Africa. Furthermore, the association of Se with selected essential and toxic elements was investigated as well as possible sex-dependent responses to Se exposure in the neonates.

This study is a part of the multi-disciplinary and multi-national research collaboration between South Africa and Norway that evaluates prenatal exposures to environmental pollutants and their effects on reproductive outcomes; the study was carried out under the auspices of the Arctic Monitoring and Assessment Program (AMAP).

## 2. Materials and Methods

### 2.1. Study Sites and Participants

**Figure 1:**
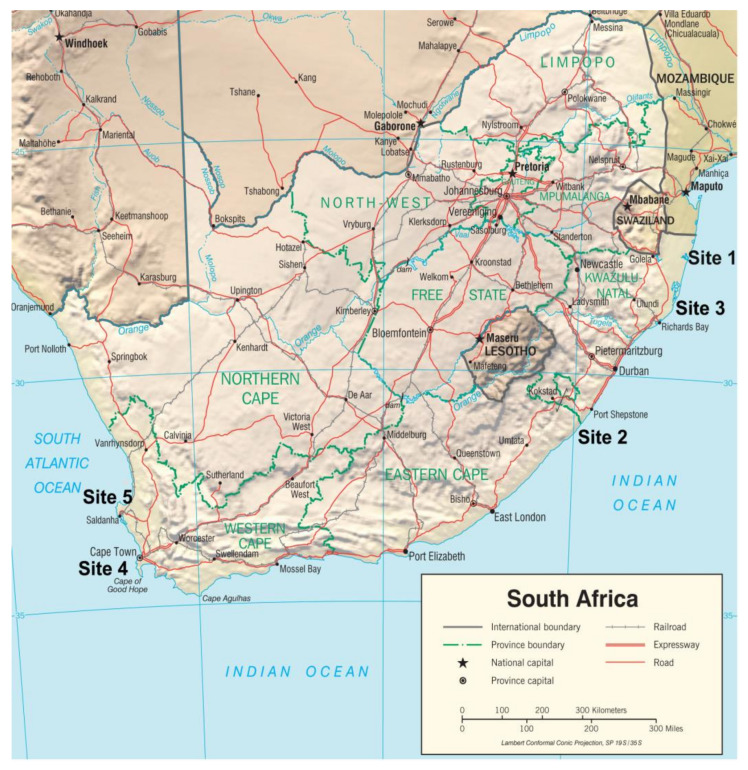
Study sites: Sites 1, 2, 3—Indian Ocean; sites 4 and 5—Atlantic Ocean. Figure is identical but site locations have been added, and is therefore for representative purposes only. https://www.cia.gov/library/publications/resources/cia-maps-publications/South%20Africa.html. (accessed on 6 March 2017).

In total, five study sites were chosen along coastal regions of South Africa (three study sites at the Indian Ocean coast of the KwaZulu-Natal (KZN) province, and two sites at the Atlantic Ocean coast of the Western Cape province) (Figure 1). All sites were rural, except for the urban study site of the city of Cape Town, in the Western Cape province. In the statistical analyses, we report the results for total cohort, Indian Ocean region, and Atlantic Ocean region, which differ by environmental pollution and socioeconomic status. Women admitted for delivery at the maternity sections of public hospitals in the study regions were informed of the study objectives by the medical personnel on duty and a research assistant and invited to participate in the investigation. They were also given a detailed information pamphlet about the study. In total, 650 women agreed to take part in the study, signed informed consent forms, and agreed to donate a blood sample before delivery. Furthermore, all study participants agreed to answer a socio-demographic questionnaire by interview, which included questions on the frequency of intake of various basic foods before and during pregnancy, lifestyle, and self-reported health status. All study participants agreed to allow researchers access to the hospital birth outcome data and understood that participation was voluntary and confidential and that they had the option of withdrawing from the study at any time.

### 2.2. Sample Collection and Analytical Procedures

A sterile Venoject system and Becton, Dickinson & Company (BD, Franklin Lakes, NJ, USA) collection tubes were used for all blood collections. Each study participant donated 10 mL of venous blood into a non-additive tube to obtain serum fractions for the analyses of Se, copper (Cu), zinc (Zn), and aluminum (Al). The serum tubes were centrifuged and the serum was transferred to acid-washed polypropylene tubes using acid-washed plastic pipettes. For the analyses of toxic elements such as Hg, lead (Pb), manganese (Mn), Cd, and As in maternal whole blood, 10 mL of venous blood was collected into tubes containing ethylene diamine tetra acetic acid (EDTA). Samples of serum (post-centrifugation) and whole blood samples were stored at −20 °C and couriered in a frozen state to the National Institute for Occupational Health (NIOH) laboratory, Johannesburg, South Africa. All precautions to eliminate and prevent contamination at collection and during the preparation of samples were applied throughout. All samples were analyzed using an Agilent Inductively Coupled Plasma Mass Spectrometer (ICP-MS) 7900 with an Octopole Reaction System. The serum samples were analyzed at Lancet Laboratories, Johannesburg, and whole blood samples were analyzed at the NIOH laboratory, Johannesburg, South Africa. Both laboratories participate in a proficiency testing scheme for biological samples.

#### 2.2.1. Serum Analyses

For the measurement of Al, Cu, Zn, and Se in serum, samples were diluted 20-fold with a diluent [ammonia 2.5 mL; butanol 6 mL, 0.1% triton-X 50 µL, and EDTA (50 µg) in 500 mL deionized water]. Ammonia, butanol, and EDTA were purchased from Merck Chemicals (PTY) Ltd., South Africa: Triton-X, calibration standards and internal standards were purchased from Industrial Analytical, South Africa. The following internal standards were also added to the diluent, indium (In, 25 µL), germanium (Ge, 25 µL), scandium (Sc, 25 µL), rhodium (Rh, 250 µL), and iridium (Ir, 250 µL). The ICP-MS instrument was calibrated with calibration standards prepared in a diluent using a multi-element custom standard (SPECTRASCAN–SS028226). The concentrations of the standards for Se, Cu, and Zn ranged from 0.1 to 100 µg/L, and for Al, the range was 0.1 to 50 µg/L. The internal standards used were Sc, Ge, Ge, and Ir for Al, Cu, Zn, and Se, respectively. The instrument was run in general purpose mode using helium gas.

Two certified reference controls, Seronorm™ Trace Elements Serum (Sero Ltd., Billingstad, Norway) were analyzed with every analytical run in intervals of 10 samples for quality assurance of all element measurements. The percentage recovery for Cu, Zn, and Se was 88–104%, and 91–114% for Al. The coefficient of variation was 8.76%, 4.56%, 4.87%, and 6.88% for Al, Cu, Zn, and Se, respectively. The limits of quantitation (LoQ) for Al, Cu, Zn, and Se were 0.15, 0.06, 0.31, and 0.17 µg/L, respectively.

#### 2.2.2. Whole Blood Analysis

The collection and analyses of samples for the selected elements manganese (Mn), Hg, Pb, Cd, and As in maternal whole blood have been described previously [40,41,42,43,44].

In short, analyses for the whole blood samples were performed on an ICP-MS, following digestion in nitric acid. Two blood certified reference controls, Seronorm ™ Trace Elements (Sero Ltd., Billingstad, Norway), were used. The percentage recovery of the Seronorm controls for the metals measured in blood ranged from 83–108%. The detection limits for Mn, Hg, Pb, Cd, and As were 0.07, 0.08, 0.04, 0.03, and 0.13 µg/L, respectively.

### 2.3. Covariates

Information on covariates was obtained from interviewer-administered questionnaires and hospital medical birth records of the neonates. Demographic and socio-economic status questions such as age, race/ethnicity, marital status, educational status, employment status, housing type, and source of water supply as well as self-evaluated health status were included in the questionnaire. Questions on nutrition of the mothers before and during pregnancy were also asked; these included the consumption of meat and fish, dairy products, and fruits. Additionally, the types of fuel used for the purpose of either cooking or heating were included in the questionnaire, as were smoking habits. Birth weight (g), birth length (cm), head circumference (cm), gestational age (weeks), Apgar score at 1 and 5 min, and placenta weight (g) were obtained from the medical records of the neonates.

### 2.4. Statistical Analyses

Data analyses were carried out using STATA version 12 [39]. The Chi-square test was used to determine if the socio-demographic and economic variables were significantly different between the two coastal regions. Bivariate analyses were carried out between Se exposure and covariates using Spearman’s correlation. All the continuous variables including maternal serum Se were found to be not normally distributed and had a skewed distribution, therefore, their median and geometric mean values were calculated rather that their arithmetic means. The Wilcoxon rank-sum test was used to compare the medians of continuous variables between the two geographical populations. For the categorical variables, missing values were treated as a separate category. Multi-variable adjusted quantile regression analysis was used to explore the risk factors associated with high Se levels in maternal serum using a backward deletion approach, starting with a full model of factors significantly associated with maternal serum Se in the univariate analysis. Statistical significance was set at *p* < 0.05 for all models.

### 2.5. Ethical Considerations

The study protocol was approved by the Human Research Ethics Committee of the University of Witwatersrand in Johannesburg (Protocol no. M10742), and by the Departments of Health of the different provinces. Personal data confidentiality and sample collection were carried out in accordance with The Code of Ethics of the World Medical Association (Declaration of Helsinki). Confidentiality was maintained by assigning identification numbers to all study participants.

## 3. Results

Although the total number of study participants was 650 (350 and 300 from the Indian and the Atlantic Ocean sites, respectively), the socio-economic, demographic, obstetrics, and birth outcome data reflected in Table 1 and Table 2 are not for all 650 participants. This is because of non-response by some of the study participants and these are shown as additional categories, which are named “missing”.

### 3.1. Characteristics of the Study Population

When examining the socio-economic and demographic characteristics of participants (Table 1) by geographical position (namely the Indian Ocean and Atlantic Ocean regional cohorts), significant differences between regions were evident for marital and educational status, ethnicity, unemployment situation, and access to electricity and the source of water. Participants from the Indian Ocean region reported better housing facilities with 83% residing in formal housing, whereas a greater number of participants from the Atlantic Ocean region had an indoor tap for water and electricity in their homes. Although folic acid is included in the vitamin supplements that are prescribed free of charge to all women attending antenatal clinics, only 45.1% of women from the Indian Ocean region reported taking them regularly, compared with 94% of women from the Atlantic Ocean region.

The dietary intake of basic food items before and during pregnancy was significantly different between the two regions for fruits, meat, and dairy products. However, there was no difference between regions for the consumption of fresh fish.

### 3.2. Obstetrics and Birth Outcomes

The descriptive data for total and regional obstetrics and birth outcomes are shown in Table 2.

The average gestational age was 38.2 weeks with 49.9% of neonates being males. In the total cohort, the median age of mothers was 24.0 years with about 44% being primiparous (results not shown). There were differences in median gestational age between the Atlantic Ocean and the Indian Ocean regions (39.0 weeks vs. 37.0 weeks; *p* < 0.001). There were no differences in the median values of maternal weight, maternal height, and birth weight, and in the categories of the Apgar scores at 5 min between the two regions. For the Apgar score at 1 min, the number of neonates with scores between 7 and 10 was higher in the Indian Ocean region than in the Atlantic Ocean region (96.8% versus 88.4%; *p* < 0.001).

### 3.3. Serum Se Levels in Mothers and Infant Anthropometry Measures at Birth

Spearman’s rank correlation coefficient (*p*-value) data of the associations between maternal serum Se and birth outcomes for total, pre-term, and full-term deliveries are presented in Table 3. No significant differences were observed between pre-term and full-term births with respect to maternal serum Se concentrations. The head circumference of neonates was negatively correlated with maternal serum Se levels in the total cohort (β = −0.164; *p* < 0.001) and in both the pre-term (β = −0.234; *p* = 0.001) and full-term neonates (β = −0.132; *p* = 0.020). However, there was no correlation between maternal serum Se and birth length, birth weight, or Apgar scores at 1 min and 5 min of all the neonates.

The main finding related to birth outcomes is that the head circumference of neonates is significantly negatively correlated with maternal serum Se levels in the total cohort (β = −0.164; *p* < 0.001) as well as in the pre-term (β = −0.234; *p* = 0.001) and full-term (β = −0.132; *p* = 0.020) cohorts. Additionally, there is a positive correlation between parity and maternal Se in the total cohort (β = 0.126; *p* = 0.005) and the full-term cohort (β = 0.125; *p* = 0.027).

### 3.4. Concentrations of Se and Selected Essential and Toxic Elements at Delivery

Table 4 shows the concentrations of Se in maternal serum in the total and regional cohorts.

It is evident that serum Se levels at delivery were considerably lower in the Indian Ocean cohort when compared with the Atlantic Ocean cohort. The concentrations of selected essential and toxic elements are shown in Table 5.

The GM (95% CI) for Se in serum for the total cohort was 65.9 µg/L, whereas the GM for Cu and Zn was 483.8 and 2423.3 µg/L, respectively. The GM concentrations of Mn and Pb in blood were found to be 15.1 µg/L and 13.2 µg/L, respectively. The GM for Al in serum was found to be 9.1 µg/L, and the GMs for Hg, As and Cd concentrations were 0.8 µg/L, 0.6 µg/L, and 0.3 µg/L, respectively.

### 3.5. Spearman’s Rank Correlation Association between Se and Measured Essential and Toxic Elements

Table 6 shows the Spearman’s rank correlation coefficient (*p*-value) of the association between Se and measured essential and toxic elements at delivery in total and regional cohorts.

As expected, significant positive correlations were found between maternal Se concentrations and Zn and Cu, which are essential elements, in all cohorts. In contrast, for Mn (an essential and a toxic element) there were negative correlations with Se in the total cohort and in the Indian Ocean region, but these were not statistically significant. The measured toxic elements in maternal blood showed negative and significant correlations between Se and Pb and As in the total cohort only. Significant positive correlations were evident between Se and both Hg and Al in the total and Atlantic Ocean cohorts. There were also positive correlations between Se and Cd in the total and Indian Ocean cohorts.

### 3.6. Maternal Serum Se and Sex-Specific Association with Essential and Toxic Elements at Delivery

Table 7 shows prenatal sex-specific Spearman’s association between maternal serum Se and measured essential and toxic elements.

Significant positive correlations were found between maternal Se concentrations and maternal Zn levels in the total and male cohorts, with a marginal significance level also observed in the female cohort. In all cohorts, maternal Cu serum was positively correlated with the maternal Se concentration. For the toxic elements, maternal blood Pb levels were negatively and significantly correlated with maternal Se concentrations in the total and male cohorts. Maternal Hg concentrations were positively associated with maternal Se in all three cohorts. Maternal Se was negatively associated with As in all three cohorts, but found to be of statistical significance only for the total and female cohorts. The Al levels, on the other hand, were positively and significantly associated with maternal serum Se levels in the total and male cohorts. However, Cd levels in blood were significantly correlated with Se levels in serum only in the total cohort.

### 3.7. Univariate and Multi-Variable Regression Analyses

Results of univariate and multi-variable quantile regression at 50% are shown in Table 8. In the univariate regression, head circumference was the only birth outcome measured that was associated with maternal Se levels, while parity and maternal age were the only obstetric outcomes that were associated with maternal Se. There was an association between maternal Se and all essential elements, except for Mn. There was also a significant association between maternal Se and dietary and supplement intake during pregnancy.

In the multi-variable regression, maternal age, serum Zn, serum Cu, and race were associated with maternal Se levels. Compared to black Africans, study participants from other racial groups were more likely to have higher maternal Se levels (Coeff: 13.505; 95% Conf. Interval: 9.735 to 17.275). Additionally, the greater the maternal age, the higher the level of maternal Se (Coeff: 0.367; 95% Conf. Interval: 0.121 to 0.612).

## 4. Discussion

The current study has determined the Se status in utero at the delivery stage in a large cohort of pregnant women, residing along both the Indian and Atlantic Oceans coastal regions of South Africa. Highly significant differences in Se levels were found between the populations with participants from the rural Indian Ocean sites presenting with lower Se levels. This finding may indicate that some socio-economic, lifestyle, and dietary practices can be contributory factors. When compared with other studies, Se levels measured in our study were similar to those reported from Spain by Navarro et al. (1996) and those reported from Malawi and Ethiopia in pregnant populations [45,46]. On the other hand, the Se mean concentrations in our South African cohort 69.0(23.7) µg/L were lower than those reported from Kuwait, North Norwegian, and Korean pregnant populations, 107.3(6.9) µg/L, 73(15) µg/L, and median 94 µg/L, respectively [47,48,49].

Studies that have investigated Se levels at different stages of pregnancy have reported the lowest Se concentrations in the third trimester of pregnancy and at delivery. For example, Liu et al. (2017) showed that median Se levels in pregnant Chinese women declined from 77.6 µg/L in the first trimester to 65 µg/L in the third trimester [50].

To date, there are no published data on human Se levels in South Africa, but research has been conducted to investigate Se status in grazing herbivores. These studies report marginal to acute Se deficiencies in parts of the KwaZulu-Natal province and in the southern coastal region of the Western Cape province [51]; coincidentally, both of these geographical areas are the coastal sites where the current study was conducted. As the participants in the current study reside in Se-deficient geographical areas of South Africa, we can presume that their Se intake during pregnancy is low, but further studies are needed to confirm this assumption. Based on this information, the risk to immune, nervous, and reproductive systems during the developmental stages cannot be ruled out in the South African population. Ongoing research has already shown that deficiency of Se in pregnant women may result in damage to these systems. For example, low serum concentrations of Se in the first trimester of pregnancy have been shown to be a predictor of low birth weight in neonates [52]. A study performed in Australia found that an excessive dietary intake of Se was associated with prolonged pregnancy. In contrast, Se levels in Japanese pregnant women were not related to pre-term births [53,54]. These findings indicate the need to investigate the effect of supplementary intake of Se not only in pregnant women, but also in the general population. Although there have been no extensive studies in South Africa on the use of dietary supplements containing Se, it is expected that the use of supplements will be less in the lower income groups. This expectation was confirmed by a nutritional study performed in South Africa that used self-administered questionnaires at two health centers at the Atlantic Ocean site. The study demonstrated that supplement users were mainly females with higher incomes [55]. Similar to our findings, the intake of dietary supplements was found to be low among black African women residing in the UK [56]. However, without more research it is difficult to conclude whether black African women have lower Se levels due to their reduced supplement and dietary intake or because of other factors such as genetic make-up [57]. When examining birth outcomes, the current study found that the head circumference of neonates was significantly and negatively correlated with maternal serum Se levels in the total cohort (β = −0.164; *p* < 0.001) as well as in the pre-term and full-term cohorts. Our literature review did not find any studies reporting an association between Se levels and neonate head circumference, suggesting the need for further investigation. No other effects of Se on birth outcomes were present in our study, although other researchers have reported an effect of Se on birth weight [58].

Essential trace elements including Se regulate and maintain various organ systems in humans and their deficiency or excess may create a number of pathological conditions, particularly during the developmental stages. In South Africa, all women attending prenatal clinics are prescribed free supplements, but we have established that not all are actually taking supplements due to various traditional beliefs and social reasons. Our study found a significant positive correlation between maternal serum Se and the levels of the essential elements Zn and Cu in all cohorts. In contrast, some studies have found a negative correlation between Se and Zn in pregnant women, but significant positive correlations between maternal Se and Cu [59,60]. This investigation found that Mn (which is both an essential and toxic element) showed a marginal negative correlation with Se in the total and Indian Ocean cohorts, but these were not statistically significant.

The protective role of Se in the exposure to environmental toxic elements was also evaluated in the current study. It is important to note that all toxic elements measured (Pb, Hg, As, and Cd in maternal blood, and Al in maternal serum) were present in all samples and their concentrations were above the detection limits of the analytical instruments. In the total cohort, a significant negative correlation was found between maternal Se and Pb levels. It is reported that the competition between Se and Pb for binding with functional bio-ligands and/or in vivo formation of lead selenide are the protective mechanisms to consider [61]. Similarly, our study found a negative correlation between Se and As concentrations in the total cohort and a positive correlation for Cd in both the total and the Indian Ocean cohorts. These findings are in agreement with Zwolak (2020), who suggested that Se can reduce As- or Cd-mediated toxicity in major organs such as spleen, liver, kidney, brain, and heart in animal models and in cell culture studies, irrespective of Se form [62]. It is postulated that Se protects against the toxicity of As and Cd either by converting these metals into biologically inert compounds and/or through the action of Se-dependent antioxidant enzymes. It is also proposed that Se reduces As toxicity by increasing the As methylation efficiency. The current study found significantly positive correlations between Se and Hg levels in the total and Atlantic Ocean cohorts. It has been reported that a primary molecular mechanism to reduce toxicity of Hg is due the ability of Hg to bind to selenide or Se-containing ligands [39]. Complexes formed in such a way are irreversible, and thus, biologically inactive. In the case of Se deficiency, the impairment of normal synthesis of selenoproteins and their expression can be anticipated.

In terms of an association with Al, the present study found a significant positive correlation with Se concentrations in the total and Atlantic Ocean cohorts. In a mouse animal model, a protective effect of Se on aluminum-induced oxidative stress in liver has been recently reported [63], and Se has also been found to be effective in preventing cardiovascular damage caused by aluminum chloride in rats.

Evidence is growing for the establishment of sex-related differences in the response to prenatal exposure to environmental toxicants and dietary intake [64,65]. These studies attribute the sex-related differences to the structure and function of the placenta. In our investigation, we found evidence that the positive correlations between Se levels and essential and toxic elements may be sex-specific. The Se levels in the male cohort were positively associated with Zn, Cu, Hg, and Al levels, but negatively associated with Pb concentrations in whole blood. In the female cohort, a positive response was found for serum Cu and blood Hg, but a negative response was evident for blood As. Reports on sex-related differences in exposure, absorption, metabolism, and detoxification support the theory of susceptibility for sex-specific metal toxicity and that male neonates have a greater dependence on their mothers’ diet during gestation. Studies regarding sex differences in susceptibility to the effects of toxic and essential elements on fetal development remain scarce and there is an urgent need for large scale studies to investigate prenatal sex-specific responses to essential and toxic metal exposures.

In the final univariate analysis model, the following parameters were positively associated with Se levels: living at the Atlantic Ocean site, increase in maternal age, and increased Cu, Zn, Hg, and Al concentrations. Head circumference and Pb level were negatively associated with Se levels. However, only the following parameters showed significant associations with Se in the multi-variable regression model: maternal age, Cu and Zn levels, and being of non-African ethnicity. In addition, Se levels were found to be positively associated with maternal age, as was also shown by other researchers who attributed this to a greater health awareness as one ages [66,67].

## 5. Conclusions

The current study investigated Se status at delivery as a measure of in utero access to selected essential trace elements, and exposure to certain toxic metals during gestation. To the best of our knowledge, this is the first study in South Africa that has evaluated Se status in the developmental stages, in a large cohort of delivering women. Our study found that Se levels were comparably lower than those reported from countries in the Northern Hemisphere, and negatively associated with head circumference of neonates, indicating the need for further investigation. Furthermore, this study has shown a significant correlation between Se and other essential trace elements such as Cu and Zn, which was also evident in the multi-variable regression model. Our study is also the first in South Africa to examine Se levels and sex-dependent responses in utero to both essential and toxic elements.

The authors are aware that the present study has a number of limitations, one being reliance on participant memory for information on diet and lifestyle. Although every effort was made to obtain reliable information such as conducting interviews in the participants’ home language, there may still have been information bias due to misinterpretation, cultural misunderstanding, or poor recall. Another limitation is the fact that for practical and logistical reasons, all participants were recruited from government hospitals and no subjects were recruited from private hospitals, introducing an economic bias to the study. The cross-sectional design of the study was also a limiting factor. The study findings will form a baseline for further investigations in other areas of South Africa as well as in the rest of Africa; the findings may inform public health and regulatory agencies in formulating reproductive health policies.

## Figures and Tables

**Table 1:** Socio-economic, demographic, and dietary characteristics of participants in the total cohort and by regional cohorts.

Characteristic	Total (*n* = 650)	Indian Ocean (*n* = 350)	Atlantic Ocean (*n* = 300)	*p*-Value
Marital status (%, *n*)				<0.001
Married	22.5 (146)	11.7 (41)	35.0 (105)	
Divorced/single/widow	58.6 (381)	71.7 (251)	43.3 (130)	
Living together	17.2 (112)	14.3 (50)	20.7 (62)	
Missing	1.7 (11)	2.3 (8)	1.0 (3)	
Education (%, *n*)				<0.001
None/Primary school	8.3 (54)	14.3 (50)	1.3 (4)	
Secondary school	62.0 (403)	44.0 (154)	83 (249)	
Tertiary	25.9 (168)	35.4 (124)	14.7 (44)	
Missing	3.9 (25)	6.3 (22)	1.0 (3)	
Race/Ethnicity (%, *n*)				<0.001
African/Black	76.6 (498)	96.6 (338)	53.3 (160)	
Other	21.1 (137)	1.1 (4)	44.3 (133)	
Missing	2.3 (15)	2.3 (8)	2.3 (7)	
Employment (%, *n*)				<0.001
Employed	23.5 (153)	13.7 (48)	35.0 (105)	
Unemployed	74.3 (483)	84.0 (294)	63.0 (189)	
Missing	2.2 (14)	2.3 (8)	2.0 (6)	
Housing type (%, *n*)				<0.001
Formal housing	60.9 (396)	81.4 (285)	37.0 (111)	
Flat	4.9 (32)	3.4 (12)	6.7 (20)	
Backyard dwelling	13.5 (88)	8.3 (29)	19.7 (59)	
Informal house (shack)	3.4 (22)	4.6 (16)	2.0 (6)	
Missing	17.2 (112)	2.3 (8)	34.7 (104)	
Water source (%, *n*)				<0.001
Indoor tap	25.9 (168)	10.3 (36)	44.0 (132)	
Outdoor tap	46.9 (305)	68.6 (240)	21.7 (65)	
Others	9.4 (61)	17.4 (61)	0 (0)	
Missing	17.9 (116)	3.7 (13)	34.3 (103)	
Gestational age (%, *n*)				<0.001
Pre-term (24–37 weeks)	31.7 (206)	44.6 (156)	16.7 (50)	
Full-term (38–44 weeks)	53.5 (348)	43.1 (151)	65.7 (197)	
Missing	14.8 (96)	12.3 (43)	17.7 (53)	
Cooking fuel (%, *n*)				<0.001
Electricity	73.7 (479)	55.7 (195)	94.7 (284)	
Paraffin	5.2 (34)	7.4 (26)	2.7 (8)	
Gas/wood	19.5 (127)	35.1 (123)	1.3 (4)	
Missing	1.5 (10)	1.7 (6)	1.3 (4)	
Heating (%, *n*)				<0.001
Electricity	44.8 (291)	38.6 (135)	52.0 (156)	
Paraffin	12.0 (78)	4.9 (17)	20.3 (61)	
Gas/wood/coal	13.4 (87)	22.6 (79)	2.7 (8)	
None	23.7 (154)	31.1 (109)	15.0 (45)	
Missing	6.2 (40)	2.9 (10)	10.0 (30)	
Prescribed vitamin supplements during pregnancy (%, *n*)				<0.001
No supplements	19.9 (129)	33.4 (117)	4.0 (12)	
Supplements	43.7 (284)	27.4 (96)	62.7 (188)	
Missing	36.5 (237)	39.1 (137)	33.3 (100)	
Meat consumption before pregnancy (%, *n*)				0.004
Seldom/At least once a week	60.5 (395)	66.3 (232)	53.7 (161)	
Almost everyday	32.9 (214)	28.6 (100)	38.0 (114)	
Missing	6.6 (43)	5.1 (18)	8.3 (25)	
Meat consumption during pregnancy (%, *n*)				<0.001
Seldom/At least once a week	58.5 (380)	66.0 (231)	49.7 (149)	
Almost everyday	30.6 (199)	27.1 (95)	34.7 (104)	
Missing	10.9 (71)	6.9 (24)	15.7 (47)	
Fresh fish consumption before pregnancy				0.001
Seldom/At least once a week	66.8 (434)	62.6 (219)	71.7 (215)	
Almost everyday	10.6 (69)	9.1 (32)	12.3 (37)	
Missing	22.6 (147)	28.3 (99)	16.0 (48)	
Fresh fish consumption during pregnancy (%, *n*)				<0.001
Seldom/At least once a week	65.4 (425)	61.4 (215)	70.0 (210)	
Almost everyday	10.6 (69)	8.6 (30)	13.0 (39)	
Missing	24.0 (156)	30.0 (105)	17.0 (51)	
Fruit consumption before pregnancy (%, *n*)				<0.001
Seldom/At least once a week	23.9 (155)	36.3(127)	9.3 (28)	
Almost everyday	65.1 (423)	48.6(170)	84.3 (253)	
Missing	11.1 (72)	15.1(53)	6.3 (19)	
Fruit consumption during pregnancy (%, *n*)				<0.001
Seldom/At least once a week	23.4 (152)	36.6 (128)	8.0 (24)	
Almost everyday	65.5 (426)	48.3 (169)	85.7 (257)	
Missing	11.1 (72)	15.1 (53)	6.3 (19)	
Consumed dairy products before pregnancy (%, *n*)				<0.001
Seldom/At least once a week	28.6 (186)	42.6 (149)	12.3 (37)	
Almost everyday	57.7 (375)	41.7 (146)	76.3 (229)	
Missing	13.7 (89)	15.7 (55)	11.3 (34)	
Consumed dairy products during pregnancy (%, *n*)				<0.001
Seldom/At least once a week	27.9 (181)	41.7 (146)	11.7 (35)	
Almost everyday	57.5 (374)	41.1 (144)	76.7 (230)	
Missing	14.6 (95)	17.1 (60)	11.7 (35)	

**Table 2:** The median of obstetrics and birth outcomes by total and regional cohorts.

Characteristic	Total (*n* = 650)	Indian Ocean (*n* = 350)	Atlantic Ocean (*n* = 300)	*p*-Value
Maternal age (y)	24.0	23.0	26.0	<0.001
Maternal weight (kg)	71.6	71.0	72.0	0.716
Maternal height (cm)	159.0	160.0	158.0	0.117
Maternal blood pressure (BP, mmHg)				
BP Systolic	-	-	120.0	
BP Diastolic	-	-	70.0	
Gestational age (weeks)	38.2	37.0	39.0	<0.001
Birth weight (g)	3100.0	3100.0	3100.0	0.295
Birth length (cm)	50.0	50.0	50.0	1.000
Head circumference (cm)	35.0	35.0	34.0	<0.001
Placenta weight (g)	-	-	600.0	
Apgar score 1 min (%, *n*)				<0.001
0–3	1.4 (9)	1.1 (4)	1.7 (5)	
4–6	5.2 (34)	1.7 (6)	9.3 (28)	
7–10	85.9 (558)	87.4 (306)	84.0 (252)	
Missing	7.5 (49)	9.7 (34)	5.0 (15)	
Apgar score 5 min				0.044
0–3	0.3 (2)	0.3 (1)	0.3 (1)	
4–6	0.9 (6)	1.1 (4)	0.7 (2)	
7–10	91.4 (594)	88.6 (310)	94.7 (284)	
Missing	7.4 (48)	10.0 (35)	4.3 (13)	
Sex (%, *n*)				0.596
Male	49.9 (324)	48.0 (168)	52.0 (156)	
Female	44.6 (290)	46.3 (162)	42.7 (128)	
Missing	5.5 (36)	5.7 (20)	5.3 (16)	
Parity (%, *n*)				0.005
0	42.0 (273)	47.7 (167)	35.3 (106)	
1+	54.5 (354)	49.4 (173)	60.3 (181)	
Missing	3.5 (23)	2.9 (10)	4.3 (13)	

**Table 3:** Spearman’s rank correlation (*p*-value) of association between maternal serum Se and birth outcomes for pre-term and full-term deliveries.

Characteristic	Total (*n* = 554)	Pre-Term (24–37 Weeks) (*n* = 206)	Full-Term (38–44 Weeks) (*n* = 348)
Birth outcome	β	β	β
Birth length	−0.030 (0.499)	−0.097 (0.187)	−0.016 (0.780)
Birth weight	0.005 (0.920)	−0.062 (0.402)	0.010 (0.855)
Apgar score 1 min	0.025 (0.576)	0.075 (0.309)	0.002 (0.970)
Apgar score 5 min	−0.052 (0.248)	−0.059 (0.419)	−0.040 (0.484)
Head circumference	−0.164 (< 0.001) ***	−0.234 (0.001) ***	−0.132 (0.020) *
Parity	0.126 (0.005) **	0.122 (0.096)	0.125 (0.027) *

* *p* ≤ 0.05, ** *p* ≤ 0.01, *** *p* ≤ 0.001.

**Table 4:** Maternal serum Se concentrations (μg/L)-total and by regional cohorts (Indian Ocean and Atlantic Ocean).

Selenium Concentrations	Total (*n* = 635)	Indian Ocean (*n* = 345)	Atlantic Ocean (*n* = 290)	*p*-Value
Se				<0.001
Mean (SD)	69.0 (23.7)	63.2 (19.5)	76.0 (26.3)	
Geometric mean (GM)	65.9	60.8	72.6	
Range	10–328.2	26.1–182.5	10.0–328.2	
95% Conf. Interval	64.4–67.5	59.0–62.6	70.2–75.2	
Median	66.2	60.5	75.0	
Range	64.5–68.2	26.1–182.5	10–328.2	

**Table 5:** Concentration of selected essential and toxic elements in serum and whole blood (as indicated) in µg/L.

Element	N*	Mean (SD)	Range	GM	95% CI	Median	95% CI
Essential elements-Se, Zn Cu, Mn levels							
Se serum	635	69.0 (23.7)	10.0–328.2	65.9	64.4–67.5	66.2	64.49–68.16
Zn serum	637	503.1 (148.7)	127.7–1810.0	483.8	473.4–494.4	486.1	477.00–497.16
Cu serum	639	2482.2 (531.0)	204.8–4453.0	2423.3	2380.5–2467.0	2417.0	2371.40–2468.87
Mn blood	636	16.3 (6.6)	2.4–43.9	15.1	14.6–15.6	15.3	14.86–15.83
Toxic elements-Pb Hg, As, Cd, Al levels							
Pb blood	640	17.7 (18.5)	0.4–316.9	13.2	12.4–14.0	14.0	13.02–15.00
Hg blood	638	1.2 (1.6)	0.2–24.2	0.8	0.8–0.9	0.7	0.67–0.79
As blood	641	0.9 (0.7)	0.07–6.2	0.6	0.6–0.7	0.7	0.67–0.74
Al serum	616	14.9 (13.5)	0.2–60.1	9.1	8.3–10.0	9.7	8.94–10.80
Cd blood	641	0.4 (0.5)	0.03–4.9	0.3	0.2–0.3	0.3	0.24–0.30

N*-Total number of samples analyzed per element differed due to missing values.

**Table 6:** Spearman’s correlation of Se and essential and toxic elements by total and regional cohorts.

Element	Total Rho (*p*-Value)	Indian Ocean Rho (*p*-Value)	Atlantic Ocean Rho (*p*-Value)
Se and essential element Zn, Cu, Mn levels-total and by region			
Zn	0.164 ( < 0.001) ***	0.362 ( < 0.001) ***	0.269 ( < 0.001) ***
Cu	0.273 ( < 0.001) ***	0.298 ( < 0.001) ***	0.338 ( < 0.001) ***
Mn	−0.053 (0.189)	−0.053 (0.332)	0.046 (0.449)
Se and toxic element Pb, Hg, As, Cd, Al levels-total and by region			
Pb	−0.163 ( < 0.001) ***	−0.029 (0.594)	0.010 (0.880)
Hg	0.205 ( < 0.001) ***	0.067 (0.216)	0.124 (0.047) *
As	−0.145 ( < 0.001) ***	0.069 (0.202)	0.055 (0.379)
Al	0.122 (0.003) **	−0.041 (0.451)	0.171 (0.006) **
Cd	0.094 (0.020) *	0.132 (0.015) *	(0.215)

* *p* ≤ 0.05, ** *p* ≤ 0.01, *** *p* ≤ 0.001.

**Table 7:** Spearman’s association between Se and sex-dependent response for essential and toxic elements at delivery.

Element	Total Cohort Rho (*p*-Value)	Male Cohort Rho (*p*-Value)	Female Cohort Rho (*p*-Value)
Selected essential elements			
Zn serum	0.164 (<0.001) ***	0.202 (<0.001) ***	0.114 (0.057)
Cu serum	0.273 (<0.001) ***	0.236 (<0.001) ***	0.323 (<0.001) ***
Mn blood	−0.053 (0.189)	−0.096 (0.091)	0.005 (0.935)
Selected toxic elements			
Pb blood	−0.163 (<0.001) ***	−0.185 (0.001) ***	−0.118 (0.053) *
Hg blood	0.205 (<0.001) ***	0.243 (<0.001) ***	0.189 (0.002) **
As blood	−0.145 (<0.001) ***	−0.092 (0.110)	−0.179 (0.003) **
Al serum	0.122 (0.003) **	0.159 (0.006) **	0.065 (0.286)
Cd blood	0.094 (0.020) *	0.105 (0.068)	0.073 (0.232)

* *p* ≤ 0.05, ** *p* ≤ 0.01, *** *p* ≤ 0.001.

**Table 8:** Coefficient estimates of quantile regression at 50% quantile-univariate and multi-variable regression.

	Univariate			Multi-Variable		
Characteristic	Coefficient	*p*-Value	95% CI	Coefficient	*p*-Value	95% CI*
Head circumference	−1.205	0.007	−2.083 to −0.327	-	-	-
Apgar score 1 min	−0.442	0.570	−1.969 to 1.085	-	-	-
Apgar score 5 min	−1.495	0.206	−3.813 to 0.823	-	-	-
Birthweight	−0.002	0.217	−0.006 to 0.001	-	-	-
Birth length	−0.250	0.346	−0.769 to 0.270	-	-	
Parity						
0	1.0					
1	4.993	0.006	1.412 to 8.574	-	-	-
Region						
Indian	1.0					
Atlantic	14.460	<0.001	11.287 to 17.633	-	-	-
Gestational age	0.178	0.707	−0.752 to 1.108	-	-	-
Maternal age	0.328	0.022	0.048 to 0.608	0.367	0.004	0.121 to 0.612
Maternal weight	0.062	0.325	−0.062 to 0.186	-	-	-
Maternal height	−0.086	0.373	−0.274 to 0.103	-	-	-
Zn	0.018	0.005	0.005−0.030	0.022	<0.001	0.111 to 0.033
Cu	0.010	<0.001	0.007 to 0.013	0.008	<0.001	0.005 to 0.011
Mn	−0.262	0.060	−0.534 to 0.011	-	-	-
Pb	−0.115	0.018	−0.211 to −0.020	-	-	-
Hg	3.264	<0.001	2.251 to 4.277	-	-	-
As	−2.118	0.092	−4.585 to 0.350	-	-	-
Al	0.368	<0.001	0.242 to 0.494	-	-	-
Cd	5.007	0.006	1.451 to 8.564	-	-	-
Location						
Rural	1.0					
Urban	12.130	<0.001	8.456 to 15.804	-	-	-
Race						
African/Black	1.0			1.0		
Other	11.730	<0.001	7.925 to 15.535	13.505	<0.001	9.735 to 17.275
Smoked during pregnancy						
No	1.0					
Yes	6.420	0.001	2.731 to 10.109	-	-	-
Consumed dairy products before pregnancy						
Seldom/At least once a week	1.0					
Almost everyday	6.402	0.001	2.567 to 10.238	-	-	-
Consumed dairy products during pregnancy						
Seldom/At least once a week	1.0					
Almost everyday	6.800	<0.001	3.000 to 10.603	-	-	-
Consumed fruits before pregnancy						
Seldom/At least once a week	1.0					
Almost everyday	7.630	0.001	3.026 to 12.235	-	-	-
Consumed fruits during pregnancy						
Seldom/At least once a week	1.0					
Almost everyday	7.540	0.001	3.043 to 12.037	-	-	-
Use of supplements						
No supplement use	1.0					
Supplement use	6.070	0.024	0.796 to 11.344	-	-	-

*CI-Confidence Interval

## Data Availability

Part of these data are still unpublished and under ongoing assessment and not available publicly yet.
